# Social friction vs. cognitive efficiency: A comparative analysis of help-seeking behaviors in human communities and generative AI

**DOI:** 10.1371/journal.pone.0348441

**Published:** 2026-04-29

**Authors:** Li Qi, Lanlan Zhao

**Affiliations:** 1 School of Humanities and Education, Jiangmen Polytechnic, Jiangmen, China; 2 Beijing Science and Technology Education Center, Beijing, China; Golestan University, IRAN, ISLAMIC REPUBLIC OF

## Abstract

Generative AI agents (e.g., ChatGPT) provide a private, low-latency environment for knowledge acquisition, distinct from public online communities where social evaluation is prevalent. This study investigates how the removal of social evaluation threats in AI interactions alters learners’ help-seeking strategies. We analyzed a matched corpus of 30,000 dialogue turns from LMSYS-Chat-1M (Human-AI) and Stack Exchange (Human-Human) using computational linguistic methods (LIWC-22 and RoBERTa). Results show that in AI interactions, learners almost completely abandon defensive impression management strategies (such as hedges) and politeness markers that are mandatory in human communities. Furthermore, contrary to the expectation that users would “confess” ignorance to AI, we found that learners adopt an authoritative “Director” stance rather than a humble “Petitioner” role. These findings suggest that AI is not merely a social substitute but a functional tool that allows users to bypass the cognitive costs of social negotiation. The shift implies a trade-off: AI maximizes information retrieval efficiency but potentially reduces the ‘desirable difficulties’ associated with problem formulation. This transformation highlights a potential for reduced practice in the cognitive processes traditionally required to structure ambiguity in collaborative settings.

## Introduction

### The evolution of “safe” learning spaces: From screen interfaces to AI agents

A decade ago, Kemp and Grieve [1] highlighted a persistent paradox in higher education: while students objectively performed well in online environments, they subjectively preferred face-to-face interaction due to its “richness.” However, their study uncovered a critical nuance that has gained renewed relevance in the age of AI: a subset of students—particularly those prone to anxiety—preferred digital interfaces precisely because they offered “less judgment.”

This observation points to a fundamental trade-off in educational psychology: the conflict between social presence and evaluation apprehension. In traditional human-to-human interaction (HHI), asking a question is rarely a neutral act of information retrieval. It is a social performance fraught with risk. According to Schlenker’s [2] self-presentation theory, learners in public settings (classrooms or forums) must constantly monitor how their questions reflect on their competence. This “social visibility” creates a cognitive burden: learners may choose to remain silent rather than risk exposing their ignorance to peers or instructors [3]. The emergence of Generative AI (GenAI) has fundamentally altered this calculus. Unlike the asynchronous Computer-Mediated Communication (CMC) context studied by Kemp and Grieve [1], modern Large Language Models (LLMs) offer a novel interactive affordance: immediate, personalized feedback without social scrutiny [4–9].

While the “Computers as Social Actors” (CASA) paradigm [10] traditionally suggests that humans unconsciously apply social norms to machines, recent developments suggest a divergence. Contemporary AI agents are increasingly perceived not merely as “social actors,” but as instrumental tools that simulate human competence while stripping away human judgment [11]. This creates a theoretical imperative to revisit the “face-to-screen” debate. When the screen is no longer a passive medium but an active, non-judgmental intelligence, does it simply reduce anxiety, or does it fundamentally restructure the social mechanisms of help-seeking?

This study investigates this shift by contrasting the high-friction social environment of human communities with the zero-friction environment of Generative AI. We posit that the “safety” provided by AI is not merely a matter of convenience, but represents a structural removal of the social costs associated with learning.

### The sociology of help-seeking: Face threats vs. algorithmic safety

To understand the shift in learner behavior, we must first dissect the sociological nature of the traditional classroom. Drawing on Goffman’s [12] dramaturgical theory, the classroom is not merely a site of knowledge transmission; it is a “front stage” where students perform competence.

In this high-stakes environment, asking a question is a double-edged sword. While it serves a cognitive function (filling a knowledge gap), it simultaneously constitutes a Face-Threatening Act (FTA) [13]. Admitting “I don’t understand” publicly exposes the learner to the risk of negative evaluation—being perceived as less intelligent or inattentive.

To mitigate these social risks, learners in human-to-human interaction (HHI) are compelled to engage in defensive impression management [3]. They allocate cognitive resources not just to formulating the question, but to “softening” it. This results in what we term a “Politeness Tax”: the mandatory use of hedges (“I guess,” “Maybe”), apologies (“Sorry to ask”), and indirect phrasing to buffer the potential loss of face. In extreme cases of evaluation anxiety, this cost becomes too high, leading to ‘defensive silence’ where learning opportunities are forfeited. Generative AI fundamentally alters this calculus by removing the social other. While the “Computers as Social Actors” (CASA) paradigm [10] suggests that humans automatically transfer social norms to machines, recent evidence points to a limit in this transfer. Lucas et al. [11] demonstrated that virtual agents, precisely because they lack the capacity for moral judgment or gossip, can elicit greater disclosure than human partners.

However, in the specific context of academic help-seeking, we propose that this “safety” manifests not as emotional intimacy, but as instrumental efficiency. Unlike a human teacher, an AI agent does not judge the quality of a question. It does not roll its eyes at repetitive inquiries, nor does it form a negative impression of the learner’s intelligence. We acknowledge that interaction norms in human communities are shaped not only by psychological evaluation apprehension but also by explicit platform governance (e.g., Stack Exchange’s requirement to demonstrate prior effort). Therefore, this study operationalizes ‘Social Friction’ as the aggregate cognitive cost imposed by both implicit social norms and explicit community guidelines. The comparison with AI highlights the removal of these external constraints. The central question of this research is: when the social necessity for impression management is removed, do learners simply become “relaxed,” or do they adopt a fundamentally different, perhaps more aggressive, epistemic role?

### The dialectics of safety: Frictionless inquiry and potential skill trade-offs

While AI alleviates immediate anxiety, a deeper sociopsychological perspective suggests that this “safety” comes with a hidden price tag. Turkle [14] warned that the allure of digital companionship lies in its ability to offer interaction that is “friction-free”—cleaned of the unpredictability and emotional demands of face-to-face encounters.

In the educational context, AI realizes Rogers’ [15] ideal of “unconditional positive regard”—but in a synthetic form. It answers every question without judgment, sighing, or fatigue. However, Caplan’s [16] social compensation hypothesis warns that when individuals with high social anxiety preferentially seek these “safe” environments, the technology transforms from a tool into a “social crutch.” This shift occurs within a broader landscape where machine learning and AI have demonstrated transformative utility across diverse and specialized domains, ranging from medical diagnostics and epidemiology [17–19] to biometric security [20–22] and engineering optimization [23–25]. However, as AI transitions from a specialized technical tool to a ubiquitous conversational agent, its impact on the sociolinguistic dynamics of human inquiry remains less understood.

From a Cognitive-Behavioral (CBT) perspective, relying on AI to bypass human interaction functions as a classic “Safety Behavior” [26]. This creates a temporal paradox: while avoidance provides immediate relief from evaluation anxiety through negative reinforcement, it simultaneously inflicts long-term damage by preventing “fear extinction.” By systematically avoiding the “scary” instructor or peer, the learner is deprived of the opportunity to learn that social risks are manageable. Consequently, the fear of human interaction is not extinguished; it is preserved and entrenched.

Recent critiques have focused on cognitive deskilling and over-reliance on external tools (e.g., students losing the ability to write) [27–29]. We extend this concern to social deskilling. If learners habituate to the “Director” stance—where communication is strictly instrumental and command-based—they risk losing the “soft skills” of negotiation, patience, and politeness required in human hierarchies. As they acclimate to the algorithmic greenhouse where every demand is met instantly, re-entering the “high-friction” reality of the classroom may trigger a rebound effect of heightened anxiety and intolerance for social ambiguity.

### The present study: From social friction to algorithmic efficiency

While a growing body of literature has assessed the impact of generative AI on learning outcomes [30] and anxiety reduction [31], recent empirical studies further indicate that AI fundamentally restructures learners’ affective states and engagement. This is particularly evident in high-anxiety domains such as foreign language learning, where AI-mediated instruction significantly shapes students’ motivational climates [32] and task engagement [33]. Moreover, interaction with multimodal AI triggers complex positive and negative achievement emotions rather than simple anxiety reduction [34], a dynamic that is heavily contingent upon the GenAI literacy of both instructors and learners [35]. Despite this recognized impact on psychological and emotional variables in specific pedagogical tasks, less attention has been paid to the fundamental shift in interaction norms during general, unstructured help-seeking. How do learners adjust their inquiry strategies when the partner shifts from a judging human peer to a non-judgmental algorithm? Traditional methods, such as self-reports, often fail to capture these subtle behavioral shifts due to social desirability bias—learners rarely admit to avoiding questions out of fear of embarrassment [36].

To address this gap, this study moves beyond self-reported data to analyze revealed preferences in natural language. We employ a computational sociolinguistic approach to contrast two fundamentally different interactional landscapes: a rule-bound, public, and meritocratic human community (Stack Exchange) versus a rule-free, private, and dyadic generative AI tool (LMSYS-Chat). This framework allows us to operationalize “Social Friction” as a composite construct arising from both psychological evaluation anxiety and codified platform governance. By comparing these two ecological niches, we aim to uncover how help-seeking strategies transform when both institutional and interpersonal constraints are systematically removed. By quantifying linguistic features (using LIWC-22) and emotional patterns (using RoBERTa) across these 30,000 matched dialogue turns, we test two competing hypotheses regarding the dynamics of social friction: (H1) The Efficiency-Politeness Trade-off (Revisiting Impression Management). We posit that “politeness” in educational settings is largely a mechanism to mitigate social friction. Consequently, in the AI environment—where social friction is structurally removed—we hypothesize that learners will abandon “social lubricants” (e.g., hedges, apologies, honorifics). This would suggest that the “safety” provided by AI is derived from de-socialization, reducing the interaction to pure information transaction and (H2) Shift in Epistemic Authority (From “Petitioner” to “Director”). Contrary to the view that learners use AI to safely “confess” their confusion, we hypothesize a reversal of roles. In human communities, help-seekers must often adopt a humble “Petitioner” stance (e.g., admitting ignorance, explaining context) to elicit help. In contrast, we expect learners interacting with AI to assume a “Director” stance, utilizing imperative commands to extract information without exposing their own cognitive vulnerability.

This study does not merely aim to prove that “people are rude to bots.” Rather, it seeks to uncover the hidden cost of efficiency: by bypassing the social negotiation required in human communities, are learners losing the opportunity to practice the resilience required for real-world collaboration?

## Literature review

### The cost of questioning: Impression management as a cognitive burden

To understand why learners migrate to AI, we must first analyze the sociological “price” of interaction in human environments. Drawing on Goffman’s [12] dramaturgical framework, the classroom—whether physical or digital—functions not merely as a site of information exchange, but as a “front stage” where competence is performed. In this context, learners are not just processing information; they are social actors operating under the continuous gaze of peers and instructors [3].

Within this high-visibility arena, the act of asking a question is structurally dangerous. It constitutes a prototypical Face-Threatening Act (FTA) [13]. By admitting a gap in knowledge (“I don’t understand”), the learner risks being categorized as “low-ability” or “lazy” [2]. This risk is amplified in meritocratic communities (like Stack Exchange or competitive seminars), where status is derived from demonstrated expertise.

To mitigate this risk, learners engage in defensive impression management. They cannot simply ask for information; they must wrap their inquiry in layers of social padding. This typically manifests in two strategies: first, the use of hedges (e.g., “I might be wrong,” “Just wondering”) to soften the imposition; and second, the construction of effort narratives (e.g., “I have tried X and Y...”), which explicitly detail prior attempts to prove they are not “free-riding” on the community.

We conceptualize these behaviors as a “Politeness Tax.” While these strategies successfully lubricate social friction, they impose a significant extraneous cognitive load. The learner is forced to split their attentional resources: processing the academic content while simultaneously monitoring their social standing. This theoretical framing suggests that the “Petitioner” role in human interaction is inherently high-friction and cognitively expensive.

### From social actors to instrumental servants

To explain how learners interact with AI, we must revisit the foundational “Computers as Social Actors” (CASA) paradigm [10]. This framework traditionally posits that humans are hardwired to apply social rules—such as reciprocity and politeness—to any entity that exhibits language, even if they know it is a machine [37].

However, the advent of Large Language Models (LLMs) challenges the universality of CASA. While modern AI agents are linguistically sophisticated, users are increasingly aware of their ontological status: AI possesses high competence (it knows facts) but lacks agency (it has no feelings or social standing) [38].

This distinction is critical. In a social interaction, politeness is a mechanism to protect the other’s feelings (Face). If the user perceives that the partner has no feelings, the functional basis for politeness collapses. Recent research suggests that as AI becomes more capable, the interaction shifts from a “social dialogue” to an “instrumental command” structure. Mollick and Mollick [5] describe this as treating AI as a “co-intelligence” or tool, rather than a peer.

This asymmetry—interacting with an entity that is “smart” but “soulless”—creates a unique affordance for help-seeking. Unlike human instructors, whose time is scarce and whose judgment matters, AI offers what we term “Asymmetrical Service.” It provides the detailed feedback of a tutor but requires none of the social reciprocity of a human.

This sets the stage for a new form of interaction that contradicts traditional classroom norms. Instead of fostering “parasocial trust” (treating the agent like a friend), we posit that learners develop “instrumental trust” (treating the agent like a search engine on steroids). This theoretical pivot explains why learners might feel comfortable abandoning the “Petitioner” role required in human hierarchies and adopting a directive stance that would be socially unacceptable with a human teacher.

### Digital safe havens: Benign disinhibition vs. social bypassing

Building on the ontological distinction between “social actors” and “tools,” we must re-examine the concept of “Benign Disinhibition.”

A substantial body of research suggests that the non-judgmental nature of virtual agents lowers the barrier for self-disclosure. In a foundational study, Lucas et al. [11] found that participants were more willing to disclose PTSD symptoms to a virtual interviewer than to a human therapist. Similarly, Gratch et al. [39] confirmed that reducing perceived social evaluation encourages users to reveal personal distress. In education, Baidoo-Anu and Owusu Ansah [31] recently observed that language learners feel safer practicing with ChatGPT because it removes the “shame” of making errors.

However, a critical distinction must be made between emotional disclosure (e.g., admitting trauma) and epistemic inquiry (e.g., asking for facts). In a therapeutic or social context, the goal is connection; therefore, “safety” leads to deeper vulnerability (“I feel sad”).

In a utilitarian learning context, the goal is efficiency. Here, we argue that the “safety” provided by AI may lead to a different outcome: Social Bypassing. Kemp and Grieve [1] noted that students prefer online environments for their “less judgment.” We posit that in the era of Generative AI, this preference evolves into a strategy of avoidance.

In human interactions, admitting ignorance (“I don’t understand this concept”) is a necessary social signal—a form of “performative humility” to validate the request for help. In AI interactions, because the agent has no social status to respect, this signal becomes redundant. Therefore, we hypothesize that “disinhibition” in this study will not manifest as more vulnerability (e.g., “I am so confused”), but as less. Learners will likely leverage the AI’s non-human nature to bypass the psychological step of admitting ignorance entirely, proceeding directly to the command (“Explain this”). This logic forms the theoretical basis for our Hypothesis H2.

### The efficiency trade-off: Frictionless interaction and potential for reduced practice

While AI alleviates immediate evaluation anxiety, clinical psychology suggests that this “safety” comes with a hidden cost. According to Cognitive-Behavioral models (CBT), avoidance strategies employed to bypass social distress are classified as “Safety Behaviors” [26].

While safety behaviors provide short-term relief (Negative Reinforcement), they are maladaptive in the long run because they prevent “Fear Extinction.” By habitually using AI to avoid the “scary” feedback of a human instructor, the learner never gathers the evidence that social criticism is manageable. Consequently, the anxiety is preserved and entrenched [40]. Sociologically, Wood [41] argues that the appeal of digital companionship lies in its “friction-free” nature. Real human interaction is messy, unpredictable, and requires constant emotional regulation. AI agents, in contrast, provide a synthetic form of “unconditional positive regard”—they respond instantly without the friction of judgment or fatigue [42].

However, Caplan’s [16] social compensation hypothesis warns that this optimization transforms technology from a bridge into a “social crutch.” For vulnerable individuals, the ease of AI interaction may displace, rather than support, human engagement. In the era of Generative AI, this risk evolves from simple “internet dependence” to “Social Deskilling.” Zeng et al. recently warned that reliance on AI could lead to cognitive deskilling (e.g., inability to write code) [27]. We extend this concern to the social domain.

If learners habituate to the “Director” stance—where every command is met with compliance—they may lose the resilience required for High-Friction human environments. This creates a potential “Rebound Effect”: students who are articulate and confident with AI may experience heightened anxiety and withdrawal when they return to the classroom and face the complexity of real-time debate, interruption, and disagreement.

### Methodological shift: Language as a trace of social power

Given that self-reported measures of anxiety are susceptible to bias—learners rarely admit to avoiding questions out of fear—this study adopts a Computational Sociolinguistic approach. We treat natural language not merely as a vehicle for information, but as a “behavioral fingerprint” of the underlying power dynamics [43]. By analyzing functional word usage, we can objectively quantify the “social friction” present in an interaction without relying on user introspection.

Drawing on Brown and Levinson’s [13] Politeness Theory, we identify specific linguistic markers that serve as the currency of social negotiation. In high-stakes human interactions, speakers must employ Hedges (e.g., *maybe*, *perhaps*, *sort of*) to reduce the certainty of their assertions, thereby mitigating the risk of conflict. Similarly, Defensive Politeness (e.g., *sorry*, *please*) acts as a repair mechanism to preempt social rejection. We posit that the density of these markers serves as a direct proxy for the intensity of the “Politeness Tax” learners feel compelled to pay.

Furthermore, the use of First-Person Singular Pronouns (*I*, *me*, *my*) offers a window into the learner’s subject position. While clinical psychology often links high “I-word” usage to anxiety or depression [44], in the context of help-seeking, we argue it reflects the “Petitioner” stance. To justify a request for help in a meritocratic community, the learner must anchor the inquiry in their own experience (e.g., “*I have tried*...”). Conversely, a reduction in “I-words”—or a shift to imperative command structures—would signal a shift toward the “Director” role, engaging in what we term Instrumental Directness.

By comparing these fine-grained linguistic features across the LMSYS (AI) and Stack Exchange (Human) corpora, this study aims to robustly validate the qualitative shift in interaction norms at a large scale, moving beyond the limitations of small-sample observation.

## Materials and methods

### Research design and data sources

To investigate the impact of social evaluation on help-seeking behaviors, we constructed a comparative corpus derived from two distinct ecological niches: a public, reputation-based human community (High Social Friction) and a private, generative AI interface (Zero Social Friction).

#### The human-human interaction (HHI) corpus: stack exchange.

Instead of using laboratory simulations, we sampled data from Stack Exchange (specifically the Academia, Psychology, and Cognitive Science sub-forums) characterized by strict community norms and reputation-based social sanctions [45–47].

While not a physical classroom, Stack Exchange represents a meritocratic epistemic community that mirrors the power dynamics of higher education. It features (1) clear status hierarchies (reputation scores), (2) strict quality standards (moderation/downvotes), and (3) public visibility. This makes it an ecologically valid proxy for “high-stakes” knowledge inquiries where impression management is crucial to avoid social sanctions (e.g., being labeled as lazy or incompetent). We extracted 25,000 initial question posts labeled as “help-seeking” (excluding meta-discussions) using the official Data Dump.

#### The human-AI interaction (HAI) corpus: LMSYS-Chat-1M.

We utilized the LMSYS-Chat-1M dataset [48], which contains real-world conversation logs between users and Large Language Models (LLMs) including GPT-4 and Claude.

Unlike synthetic data generated by researchers, this dataset captures “in-the-wild” user behaviors. The interaction is dyadic, anonymous, and functionally devoid of social judgment, representing a “safe haven” for cognitive inquiry. To ensure functional comparability with the Stack Exchange dataset, we applied a strict keyword-based filtering protocol to the LMSYS-Chat-1M corpus. We retained prompts containing epistemic intent markers (e.g., ‘explain’, ‘how to’, ‘why is’, ‘code for’, ‘what is the difference’). We explicitly excluded prompts categorized as: (1) Creative Writing (e.g., ‘write a poem’, ‘continue the story’); (2) Role-play without information retrieval intent (e.g., ‘pretend you are’); and (3) Phatic communication (e.g., ‘hi’, ‘are you there’). This resulted in a subset of prompts focused on problem-solving and information acquisition. We randomly sampled 25,000 first-turn prompts to match the initiation phase of the HHI questions.

### Semantic alignment and structural matching

A major challenge in comparing forum posts with AI prompts is the potential confounding factor of topic divergence (e.g., coding bugs vs. essay writing). To ensure we were comparing “apples to apples,” we implemented a rigorous two-step alignment protocol:

#### Topic alignment via BERTopic.

We employed BERTopic [49], a transformer-based topic modeling technique, to map both corpora into a unified semantic space. We filtered out topics exclusive to one modality (e.g., “Server Configuration” in SE or “Creative Storytelling” in AI). We retained only overlapping epistemic topics, such as Conceptual Explanations, Academic Writing Advice, and Theoretical Inquiries. This ensures that observed linguistic differences stem from the interaction partner (Human vs. AI), not the subject matter.

#### Controlling for complexity: Propensity score matching (PSM).

Following the keyword filtering and topic alignment, the initial pool consisted of 25,000 Stack Exchange posts and 25,000 LMSYS prompts. Forum posts tend to be longer than AI prompts. To rule out text length as a confounder for linguistic density, we applied Propensity Score Matching (PSM). We matched samples based on (1) Word Count (text length) and (2) Flesch-Kincaid Grade Level (cognitive complexity). Using 1:1 nearest neighbor matching with a caliper of 0.05 standard deviations, we generated a final balanced dataset of ***N*** = 30,000 texts (15,000 pairs). During this process, 20,000 samples (10,000 from each modality) were excluded because they did not have a suitable match within the specified caliper. Post-matching diagnostics showed negligible differences (Standardized Mean Difference < 0.01) in length and complexity between groups. This rigorous control allows us to attribute linguistic variations specifically to social psychological factors. By matching for Word Count and Flesch-Kincaid Grade Level, we ensured that the comparison was between texts of similar cognitive complexity. This control helps mitigate the risk that observed differences are merely a function of user expertise (e.g., experts providing brief commands versus novices providing lengthy explanations).

### Measures: Quantifying “social friction”

We operationalized “Impression Management” and “Cognitive Vulnerability” using a hybrid approach combining dictionary-based psychometrics (LIWC-22) and deep learning-based emotion detection (RoBERTa) [50–52].

#### Indicators of defensive impression management.

Drawing on Politeness Theory [13], we measured linguistic markers used to mitigate face threats: Hedges (LIWC: Tentat) – “*Words like maybe*”, “*perhaps*”, “*I guess*”. In human interaction, these soften assertions to avoid conflict. A decrease in AI interaction would signal reduced fear of error; Politeness Markers (Custom Dictionary): “*Words like please*”, “*sorry*”, “*thank you*”. We quantified the “social tax” users pay to grease interpersonal gears.

#### Indicators of epistemic stance (the “director” vs. “petitioner” shift).

To test H2, we analyzed how users position themselves relative to the knowledge source: Cognitive Vulnerability Disclosure – A composite metric tracking explicit admissions of confusion (e.g., confused, don’t understand, fail); Imperative Density – We analyzed the syntactic structure of sentence openings (trigrams) to detect command-based patterns (e.g., “*Give me*...”, “*List the*...”) versus narrative-based patterns (e.g., “*I am trying to*...”).

#### Affective cost analysis.

We fine-tuned a RoBERTa-base model on the GoEmotions dataset to detect subtle emotional signals. Unlike simple sentiment analysis (Positive/Negative), this model identifies specific social emotions relevant to learning, specifically Anxiety and Remorse (social shame), allowing us to visualize the “emotional labor” required in each context.

### Statistical analysis strategy

Data analysis was conducted using Python (SciPy and Pingouin libraries). Given the large sample size (*N* = 30,000) and the inherent distributional differences between human and AI-generated texts, our analytical strategy prioritized robustness over standard parametric assumptions.

#### Hypothesis testing and effect sizes.

To test H1 and H2, we employed Welch’s t-tests instead of Student’s t-tests. This decision was driven by the unequal variances observed between the two groups (AI responses typically exhibit lower variance than human discussions). Statistical significance was defined at *α* = .05. However, given the high statistical power associated with large datasets—where trivial differences can yield significant p-values—we adopted a dual-criterion protocol: a hypothesis was supported only if the difference was both statistically significant (*p* < .05) and substantively meaningful (Cohen’s *d* ≥ 0.2), following the guidelines by Sullivan and Feinn [53].

#### Robustness checks for skewed data.

Linguistic data (especially emotion scores from RoBERTa) often exhibit zero-inflation and heavy tails. To ensure the reliability of our findings, we implemented two verification steps: Winsorization – Extreme outliers (beyond the 99th percentile) were winsorized to prevent single data points from distorting group means; Non-parametric Bootstrapping – For the deep learning emotion probabilities (which do not follow a normal distribution), we constructed 95% Bias-Corrected and Accelerated (BCa) confidence intervals based on 1,000 bootstrap resamples. We consider the differences robust only if the 95% CIs of the two groups do not overlap.

### Ethics statement

Ethical review and approval were waived for this study due to the nature of the research involving the analysis of publicly available, de-identified datasets (LMSYS-Chat-1M and Stack Exchange Data Dump), which does not constitute human subjects research requiring IRB oversight.

## Results

Analysis of the propensity score-matched dataset (*N* = 30,000) reveals a fundamental structural divergence in how learners engage with knowledge sources. By triangulating lexical frequencies (LIWC), syntactic patterns (Trigrams), and latent topics (BERTopic), we observed that the removal of social evaluation does not merely “relax” communication; it fundamentally alters the genre of inquiry—from socially situated negotiation in human communities to instrumental command in AI interactions.

### Semantic landscape: Process inquiry vs. product generation

**Fig 1:**
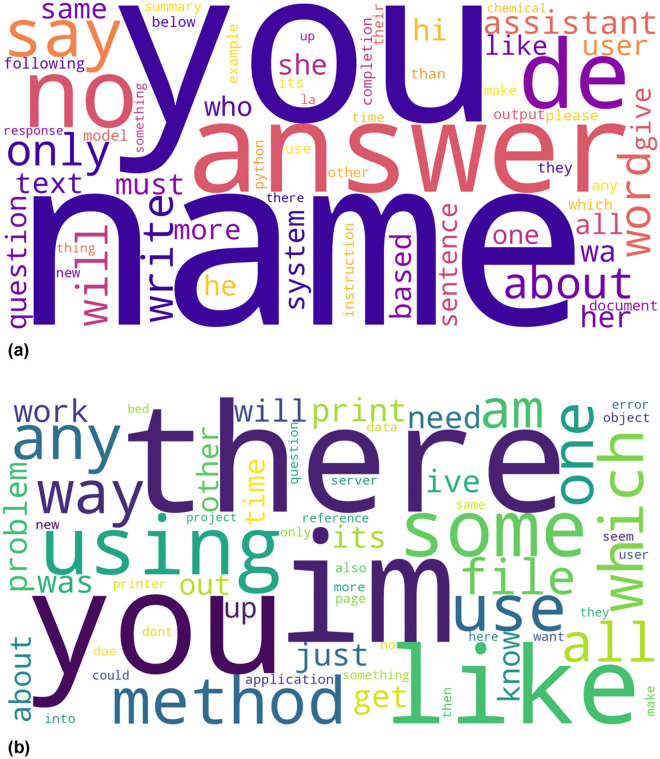
Semantic landscapes of cognitive labor: The shift from process inquiry to product generation. **(a)** AI prompts (Upper) are dominated by imperative verbs (*answer*, *write*, *give*) and output specifications (*text*, *sentence*), reflecting an “Instrumental Director” stance. **(b)** Human questions (Lower) are grounded in procedural nouns (*Method*, *Problem*, *Way*) and evidence markers, reflecting a “Struggling Petitioner” stance grounded in friction.

**Fig 2:**
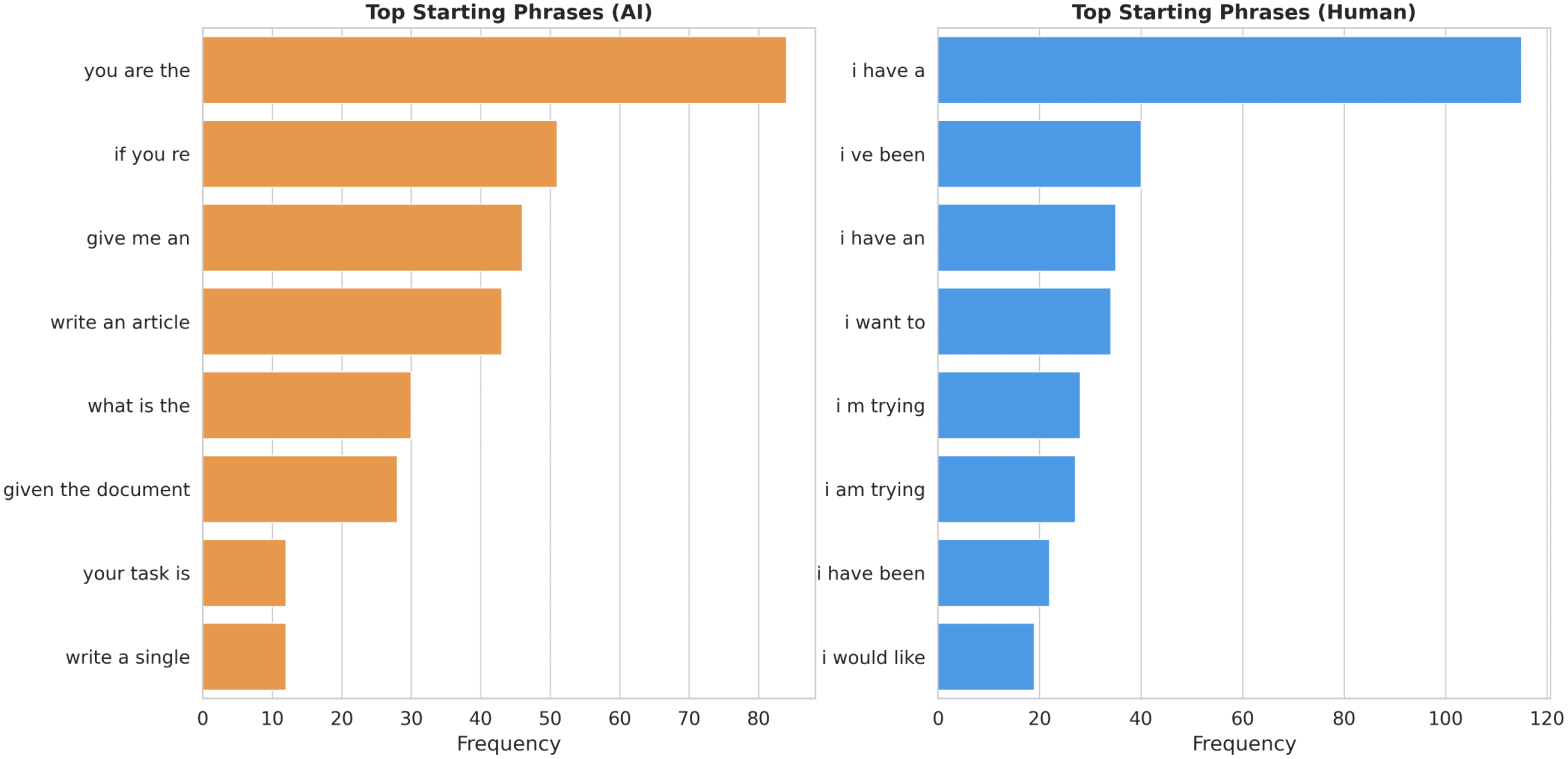
Syntactic markers of role displacement. **(a)** AI interactions (Left) prioritize ontological definition (“You are...”) and direct command (“Give me...”), establishing immediate epistemic authority. **(b)** Human interactions (Right) rely heavily on autobiographical narratives (“I have...”, “I am trying...”) to contextualize the request and validate user effort.

The semantic triangulation (Fig 1 and Fig 2) reveals a fundamental divergence in the nature of the task assigned to human peers versus AI agents.

#### The “troubleshooter” vs. the “executor”.

The word clouds illustrate a clear dichotomy between “discussing a process” and “demanding a product.” HHI (Human Context, Fig 1b): Procedural Complexity. The human semantic space is dominated by nouns related to methodology and friction. The most prominent terms include Method, Problem, Using, Way, and Time. This indicates that human-human interaction is process-oriented. Users approach the community when they are stuck in a procedure (e.g., “The method I am using is not working”). The prominence of Using and Work confirms that the interaction is grounded in an ongoing, often struggling, operational context. The user seeks a diagnosis of their process. HAI (AI Context, Fig 1a): Direct Execution. In stark contrast, the AI semantic space is saturated with directive prompts and deliverables. The visual center is heavily occupied by the pronoun “you”, action verbs (*answer*, *write*, *give*), and system roles (assistant, system). This signifies a strictly product-oriented stance. Users do not discuss the “method” of a problem; they simply command the AI to give an answer or write a text. By treating the AI as an instrumental entity rather than a collaborative peer, learners bypass the procedural struggle entirely, shifting from “learning how to do it” to “getting it done.”

#### Syntactic framing of authority.

The analysis of starting trigrams (Fig 2) corroborates this semantic shift, revealing how users construct their authority. HHI (Fig 2b): The Narrative of Struggle (“I have...”). In Fig 1b, the contraction “*Im*” (“*I am*”) appears as a dominant keyword. This aligns with the trigram data in Fig 2b, where top phrases are intensely self-referential: “*i have a*”, “*i ve been*”, “*i am trying*”. To legitimize a request for help with a Problem or Method (as seen in the word cloud), the petitioner must construct a narrative of effort. It is not enough to ask a question; one must prove they have “tried” and “been” working on it. The “Self” is presented as a struggling subject needing assistance. HAI (Fig 2a): The Assertion of Control (“You are...”). In Fig 1a, the dominance of the pronoun “you” alongside explicit commands (answer, write) presupposes a compliant subordinate. This aligns with Fig 2a, where prompts open with definitions of the other: “*you are the*”, “*act as a*”. The “narrative of effort” is entirely discarded. Instead of proving their own worthiness, the user immediately exercises ontological control over the agent. The user defines the AI’s identity and assigns the task, shifting the power dynamic from a “Petitioner” seeking guidance to a “Director” allocating labor.

### Testing H1: The dissolution of defensive norms

We analyzed the distributional properties of linguistic features to test whether AI interaction merely reduces defensiveness or fundamentally alters the structure of communication.

#### Hedges and politeness: The removal of the “social tax” (Fig 3).

**Fig 3:**
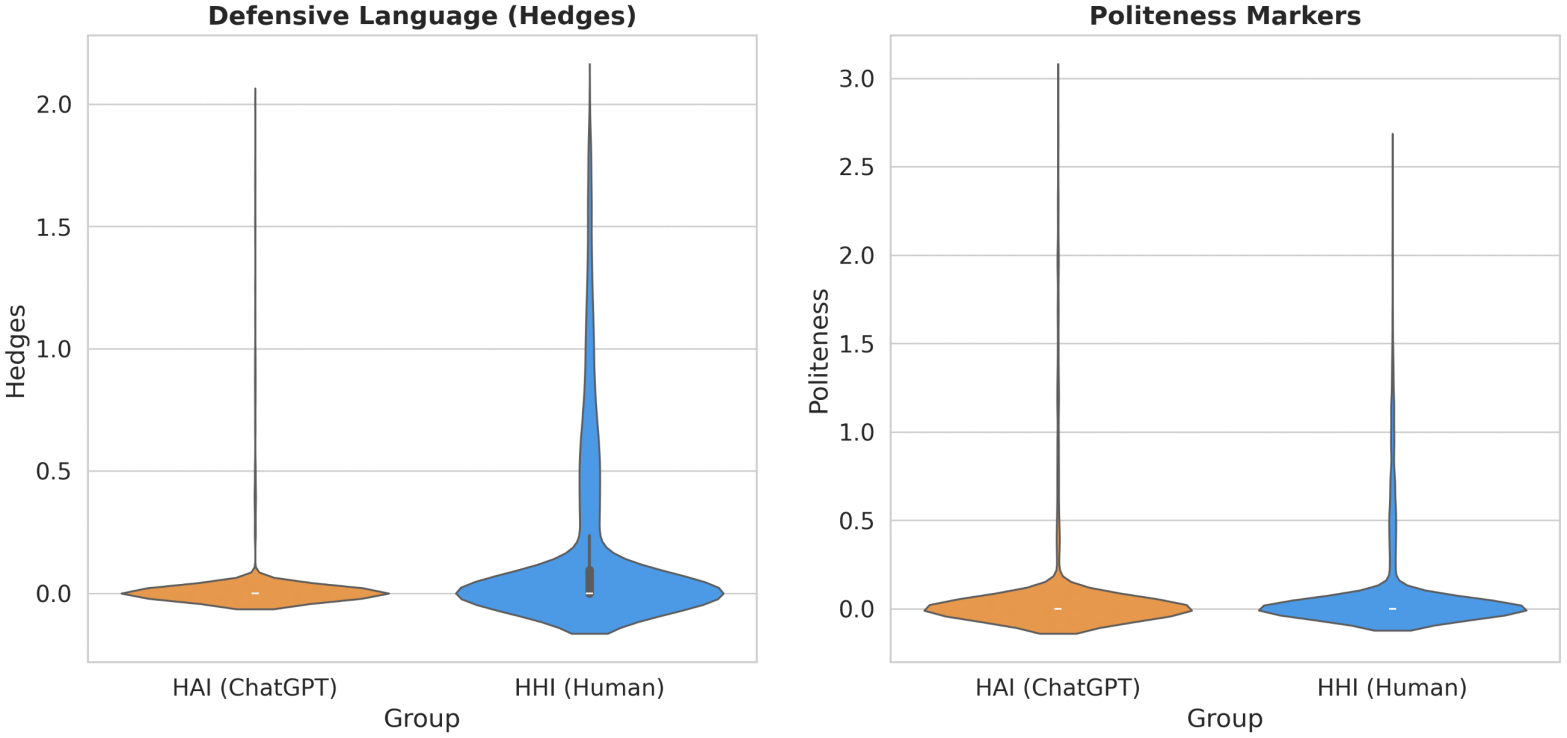
The structural dissolution of defensive social norms. **(a)** Distribution of Hedges: Human interactions (Blue/Right) maintain a normative spread, whereas AI interactions (Orange/Left) show absolute convergence at zero. **(b)** Distribution of Politeness Markers: The contrast between the “mandatory baseline” in human groups and the “zero-inflation” in AI groups confirms the removal of the “Politeness Tax” in algorithmic environments.

The violin plots in Fig 3 illustrate a transition from “mandatory social maintenance” to “efficiency-driven minimization.”

HHI (Human, Blue): The Normative Baseline. In the human control group (the blue violins on the right side of each plot), both Hedges and Politeness markers display a “bottom-heavy” distribution with a consistent baseline. The shape indicates that these linguistic features function as a mandatory entry cost. To participate in the Stack Exchange community, users effectively pay a “politeness tax”—using hedges (maybe, seems) to soften assertions and markers (thanks, please) to mitigate the imposition of asking.

HAI (AI, Orange): Zero-Inflation. In contrast, the AI distributions (the orange violins on the left side of each plot) exhibit extreme zero-inflation, collapsing into a single spike at *y* = 0. The vast majority of AI prompts contain exactly zero hedges and zero politeness markers. This confirms H1: without the threat of social sanction, learners perceive these “social lubricants” as functionally redundant. They strip the query down to its logical core, prioritizing informational efficiency over interpersonal face-saving. This represents an enormous effect size (*t*(29998)=134.52, *p* < .001, *d* = 1.63), suggesting that the compared constructs may no longer be functionally equivalent across contexts. In human forums, a hedge is a critical social signal for managing face threats; however, in the “command economy” of AI interactions, such markers are rendered not only redundant but logically incompatible with the genre of direct instruction.

#### Self-focus: The loss of behavioral constraints (Fig 4).

**Fig 4:**
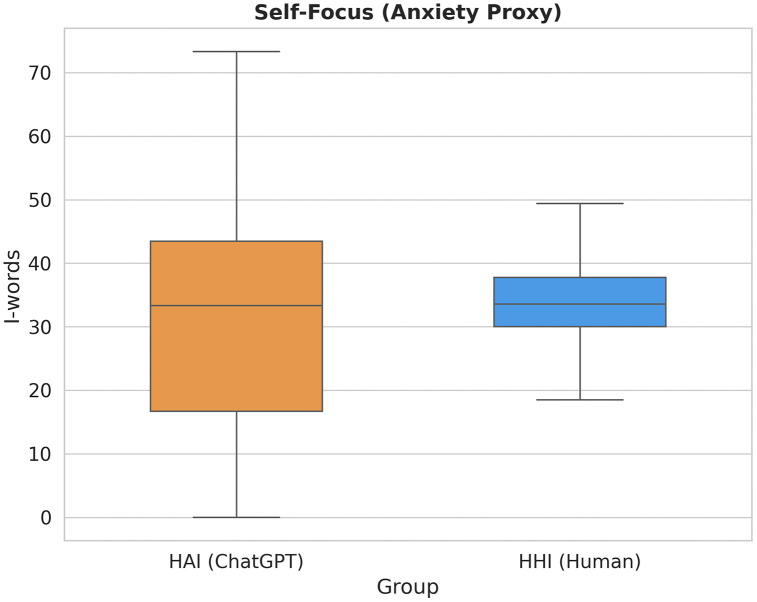
Variance in self-focus as an indicator of social deregulation. The compact Interquartile Range (IQR) of the HHI group (Blue) indicates tight social norms constraining self-presentation. In contrast, the elongated IQR of the HAI group (Orange) reveals a polarization between “silent commanders” (zero self-focus) and “confessional users” (high self-focus), characteristic of an environment lacking social supervision.

The analysis of first-person pronouns (“I-words”) in Fig 4 reveals a counter-intuitive finding regarding behavioral variance.

HHI (Human, Blue): Constrained Presentation. The human group exhibits a compact Interquartile Range (IQR). This suggests that self-presentation in public forums is governed by tight social norms: users must introduce their context (“I tried X...”), but they cannot be excessively narcissistic or purely impersonal. The community norms “clip” the extremes of behavior.

HAI (AI, Orange): Polarization and Deregulation. The AI group shows a dramatically elongated box plot with high variance. This indicates a state of social deregulation. Without normative constraints, user behavior polarizes: The Commanders – Users who eliminate “I” entirely (bottom whisker at 0), issuing pure imperatives (“Translate this”); The Confessors – Users who use “I” at extremely high frequencies (top whisker), treating the AI as a private diary or therapist.

Our data reveals that AI does not simply “reduce” self-focus, but rather facilitates a state of “Social Deregulation.” As shown in Fig 4, the HAI group exhibits significant polarization compared to the tightly constrained HHI group. While a majority of users adopt the “Director” stance (approaching zero I-words), a substantial subset occupies the opposite extreme—the “Confessors.” These users employ first-person pronouns at frequencies far exceeding human community norms, likely leveraging the AI’s non-judgmental nature for deeply personal or therapeutic disclosure. In the absence of a “social gaze” that enforces a standard “Petitioner” role, user behavior diverges into two distinct modes: purely instrumental command and radical self-expression.

#### Internal structure: Norm-driven vs. trait-driven strategies (Fig 5).

**Fig 5:**
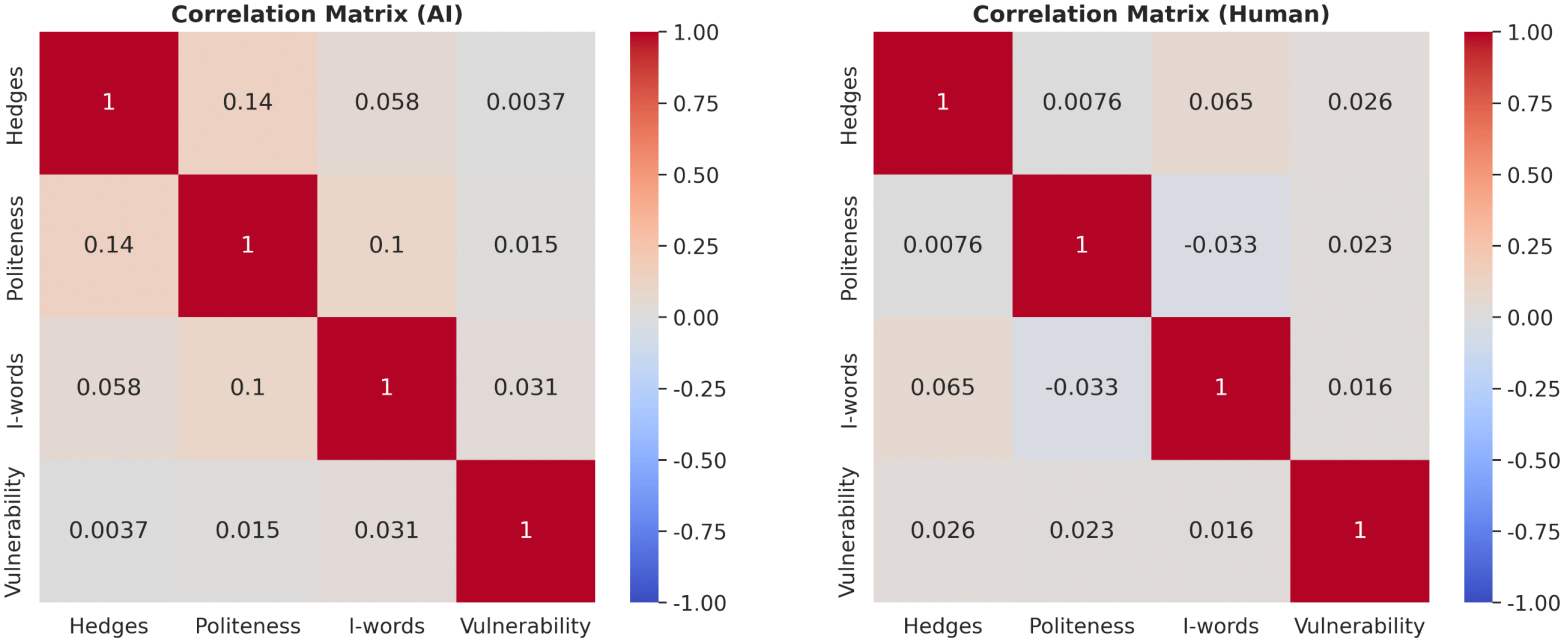
The internal consistency of impression management strategies. **(a)** The HAI group (Left) exhibits small but detectable positive correlations among social markers, reflecting a slight behavioral consistency in the absence of external constraints. **(b)** The HHI group (Right) displays near-zero correlations (“white noise”), indicating that impression management is a “norm-driven” strategic module deployed only when socially required.

The correlation matrices (Fig 5) explain why these behaviors occur by revealing the internal consistency of impression management.

HHI (Human, Right): Strategic Independence (Norm-Driven and Platform Compliance). The near-zero correlation in the HHI group indicates that linguistic strategies are employed independently based on functional requirements and explicit community guidelines rather than coherent user personality traits. Crucially, this pattern reflects an environment governed by codified platform rules (e.g., Stack Exchange’s strict requirement to demonstrate prior effort and maintain civility to avoid downvotes). Consequently, users deploy these markers as discrete tools to comply with an institutionalized checklist—adding a ‘Hedge’ to address technical uncertainty, or an ‘effort narrative’ to satisfy moderation standards. They are, essentially, checking boxes on a socio-technical protocol list to ensure their question survives community scrutiny.

HAI (AI, Left): Trait Coherence (Trait-Driven). In the AI group, while the correlation coefficients are statistically weak (ranging from *r* = 0.10 to 0.14), they represent a detectable departure from the near-zero or negative correlations observed in the HHI group. This suggests a subtle shift toward behavioral consistency; without the “decoupling” effect of platform rules, user usage of hedges, politeness, and self-reference tends to drift together slightly. While these correlations explain only a small fraction of the variance and should not be over-interpreted as strong “trait-driven” behavior, they highlight the absence of the situational pressure that characterizes human meritocratic forums.

### Testing H2: Concealment of vulnerability and social bypassing

**Fig 6:**
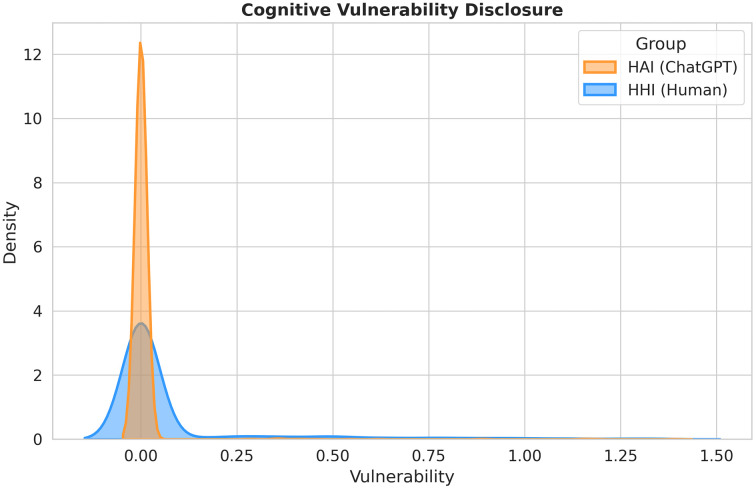
The distribution of cognitive vulnerability disclosure. The Human group (Blue) displays a “performative humility” tail (*x* > 0), reflecting the social necessity of admitting ignorance to legitimize help-seeking. The AI group (Orange) exhibits a leptokurtic peak at zero, confirming an “Instrumental Directness” where learners bypass the admission of cognitive gaps.

Hypothesis H2 predicted that the non-judgmental nature of AI would encourage learners to openly disclose their cognitive gaps (e.g., admitting confusion). However, the data in Fig 6 reveal a pattern that contradicts the “Benign Disinhibition” effect often cited in HCI literature.

#### The distribution of ignorance (Fig 6).

The Kernel Density Estimation (KDE) plot in Fig 6 visualizes a fundamental difference in how learners present their own lack of knowledge.

HHI (Human, Blue): Performative Humility. The human distribution displays a distinct right-skewed tail extending into the positive region. This confirms that in meritocratic communities like Stack Exchange, admitting vulnerability is a strategic necessity. To avoid being perceived as demanding or entitled, petitioners must perform “humility”—explicitly acknowledging their deficits (e.g., “I am totally lost,” “I fail to understand”) to legitimize their request for expert time.

HAI (AI, Orange): Instrumental Directness. In contrast, the AI distribution exhibits an extreme peak concentrated at zero. The vast majority of users completely omit markers of confusion or failure. They do not say “I don’t understand X”; they simply say “Explain X.” The statistical difference is substantial (*d* = −0.82), indicating that in the AI context, the social pressure to self-deprecate is entirely absent.

#### Mechanism: From “safety” to “social bypassing”.

Integrating these findings with the syntactic analysis in Fig 2, we propose that AI does not function as a “safe space” for emotional disclosure, but rather as a mechanism for Social Bypassing.

In Human Interaction (The Petitioner Role): The learner must adopt a low-status position. They are required to expose their “wounded” cognitive state (“I am confused”) to elicit a cure from the community. This process involves high social friction and requires emotional labor. In AI Interaction (The Director Role): The learner bypasses this negotiation entirely. Because the AI is perceived as an instrument rather than a social judge, there is no functional need to justify one’s ignorance. The learner shifts directly to the solution, adopting a high-status “Director” role.

The results suggest that the primary psychological appeal of AI in education is not that it allows students to be vulnerable without judgment, but that it allows them to be demanding without social cost. By removing the need for impression management, AI enables a form of frictionless inquiry that is efficient but socially sterile.

### Affective dynamics: Emotional labor vs. neutrality

**Fig 7:**
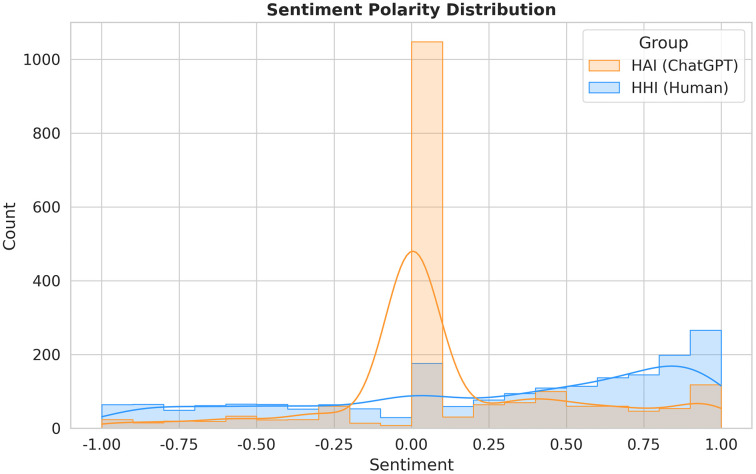
Affective dynamics and emotional labor. The HHI distribution (Blue) shows a high-positivity tail (*x* > 0.75), quantifying the “facework” required to maintain community relations. The HAI distribution (Orange) is anchored at neutral (0.0), illustrating the affective sterility of frictionless inquiry.

Fig 7 presents the distribution of sentiment polarity, quantifying the distinct emotional requirements of each interaction modality.

#### The cost of human interaction: Emotional labor (Fig 7, Blue).

The HHI distribution (Blue) is characterized by high variance and a notable density in the extremely positive region (*x* > 0.75). In a problem-solving context (e.g., fixing a bug or understanding a theory), extreme positivity rarely reflects genuine “joy.” Instead, it reflects Emotional Labor. To mitigate the friction of asking for help, human petitioners must engage in “Facework”—wrapping their requests in high-intensity gratitude (“Great insight,” “Perfect solution,” “Thanks a million”) to maintain community relationships. The broad spread indicates that human interaction requires users to constantly regulate their affect, oscillating between frustration (negative tail) and mandatory politeness (positive tail).

#### The efficiency of AI interaction: Affective neutrality (Fig 7, Orange).

The HAI distribution (Orange) exhibits a massive, narrow spike centered at 0.0 (Neutral). This confirms that AI interaction is affectively neutral. Users strip away both the frustration (negative) and the social pleasantries (positive). The interaction is reduced to a purely functional exchange of information. The absence of variance suggests that users do not feel the need to “manage” the AI’s emotions or “perform” gratitude.

The contrast between the dispersed blue curve and the spiked orange curve visually represents the “Emotional Labor Gap.” Interacting with humans imposes a tax of affective regulation; interacting with AI allows for total emotional detachment.

## Discussion

Our analysis of 30,000 interaction logs reveals a fundamental trade-off at the heart of the digital learning transition. While Generative AI successfully mitigates the anxiety associated with public help-seeking, it does so by fundamentally restructuring the epistemic role of the learner. We find that the “safety” provided by AI is not a psychological buffer for vulnerability, but a structural bypass of social negotiation.

### The “politeness tax” and social bypassing

Our findings indicate that the linguistic divergence between HHI and HAI is driven by the removal of both psychological and institutional constraints. It is critical to recognize that the high density of ‘politeness’ and ‘effort narratives’ in Stack Exchange is not solely a product of internal evaluation anxiety; it is deeply intertwined with explicit platform governance. In such meritocratic forums, these linguistic markers function as mandatory compliance mechanisms to navigate strict moderation rules and avoid punitive actions (e.g., downvotes or post closures). Therefore, the ‘Politeness Tax’ we observe is a composite burden—a blend of psychological face-saving and codified institutional compliance. Generative AI eliminates the functional necessity of these markers entirely. The absence of hedges and effort narratives in AI prompts suggests that when the requirement for community validation is removed, users default to a high-efficiency, low-maintenance communication style.

The AI interface effectively repeals this tax. The structural collapse of politeness markers (Fig 3) and the disappearance of self-justification narratives indicate that learners engage in Social Bypassing. The enormous effect size observed for hedges (*d* = 1.63) underscores a fundamental shift in the nature of the interaction. As suggested by the lack of construct equivalence across these contexts, a hedge in a human forum—meant to mitigate social friction—cannot exist in the same way within a list of AI instructions. When the interactional genre shifts from “communal discourse” to “functional command,” social lubricants are discarded not just to reduce anxiety, but because they have lost their semiotic purpose in a de-socialized, algorithmic environment. Consequently, users do not “open up” to the AI; they simply utilize it. This explains the “Zero-Vulnerability” pattern observed in Fig 6. By removing the need for impression management, AI allows learners to be socially “lazy,” bypassing the constructive friction of persuasion to access immediate information.

It is necessary to contextualize this finding of ‘affective neutrality’ and ‘social bypassing’ by comparing it with recent research on GenAI in domain-specific learning environments. While our cross-sectional analysis of general help-seeking (e.g., coding, conceptual queries) indicates emotional detachment, recent studies examining sustained educational tasks—such as EFL writing—demonstrate a contrasting pattern. In structured pedagogical settings, generative AI has been found to actively shape learners’ motivational climates and task engagement [32,33]. Furthermore, interacting with multimodal AI technologies in specific disciplines elicits complex positive and negative achievement emotions [34], rather than the pure affective neutrality observed in our dataset. This divergence suggests that the emotional valence of AI interactions is highly task-dependent. When utilized as a rapid troubleshooting tool (as seen in the LMSYS corpus), AI fosters instrumental detachment; however, when integrated into structured curriculum activities, it acts as a mediator of complex academic emotions. Navigating this boundary in practice requires a high level of GenAI literacy among both educators and students [35] to ensure that the efficiency of social bypassing does not undermine deeper emotional and cognitive engagement.

### From “process inquiry” to “product command”

The divergence in semantic focus (Fig 1) marks a critical shift in how learners conceptualize knowledge acquisition. In the human context, the dominance of nouns like Method, Problem, and Way signals that learners are positioned as Petitioners. They are engaged in a process-oriented dialogue, forced to articulate the “how” and “why” of their confusion to solicit a diagnosis.

Conversely, the AI context promotes a role reversal characterized by behavioral polarization. While the dominant trend is the ascent to the “Director” role—saturated with imperative verbs and product-oriented demands—we must also acknowledge the emergence of the “Confessor” role. For some users, the removal of social friction does not lead to authoritative command, but to an unprecedented level of vulnerability. This dual-use pattern suggests that AI functions as a “socially sterile” mirror: it reflects and amplifies the user’s immediate epistemic or emotional needs without the moderating influence of communal expectations. As they exercise this epistemic privilege, the interaction shifts from diagnosing a process to demanding a product. A Director does not need to admit confusion; they only need to specify the output. This role reversal suggests that AI is not functioning as a tutor that guides the learner through difficulty, but as a contractor that executes the labor.

Furthermore, while the “Director” stance represents the dominant behavioral shift, the high variance in self-focus (Fig 4) and cognitive vulnerability (Fig 6) suggests a secondary, albeit significant, interactional mode: the “Confessor.” For a minority of users, the removal of social friction does not lead to authoritative command, but to a radical form of self-disclosure. This pattern may reflect a therapeutic use case, where the AI’s non-judgmental nature provides a “safe haven” for individuals—particularly deeply uncertain novices or those experiencing high evaluation anxiety—to externalize their confusion without the fear of being labeled “low-ability.” In this sense, the AI functions as a socially sterile “mirror” that accommodates two extremes of the human epistemic experience: the desire for efficient, power-based control (the Director) and the need for radical, unfiltered vulnerability (the Confessor). Acknowledging this dual-use pattern implies that the “safety” of AI is not a monolith; it facilitates different strategies depending on whether the user prioritizes task optimization or psychological decompression.

### Potential risks for problem formulation skills

The shift to a ‘Director’ stance suggests a significant change in the cognitive demands of the query process. In traditional human interactions, the necessity of articulating the ‘Problem’ and ‘Method’ compels the learner to engage in cognitive scaffolding—systematically structuring their ignorance to make it intelligible to a human partner. In the AI context, however, the ability to obtain structured outputs via context-free commands (e.g., ‘give me the answer’ or ‘write a sentence’) removes this external pressure for self-explanation and scaffolding. While our cross-sectional behavioral data cannot confirm a decline in underlying cognitive capacity, the observed omission of problem-structuring language highlights a potential risk regarding the reduced frequency of practice in articulating complex epistemic gaps. Learning scientists have long posited that ‘desirable difficulties’—such as the effort required to formulate a question or explain a misconception—are essential for deep encoding. In our human data, the necessity of explaining a Problem or describing a Method forces the learner to structure their own ignorance.

In the AI context, this scaffolding is removed. The ability to obtain structured outputs via context-free commands (e.g., “give me the answer” or “write a sentence”) may allow learners to offload not just the computational task, but also the cognitive effort required for problem structuring. While this increases retrieval efficiency, it raises questions about whether the long-term omission of such ‘desirable difficulties’ could impact the development of robust problem-formulation skills. If education becomes purely product-oriented—focused on getting the answer rather than negotiating the meaning—we risk producing students who are adept at commanding information but unskilled at constructing knowledge. This concern aligns with recent discussions on “prompt engineering” as a form of cognitive scaffolding. While structured prompting can maintain explanatory quality in AI-mediated learning [54], the premature removal of human-to-human negotiation may inadvertently reduce the students’ opportunities to develop independent problem-structuring competencies.

### Re-imagining the classroom: A functional division of labor

These findings suggest that treating AI as a simple substitute for human interaction is a category error. Because AI interaction is different in kind—characterized by a fundamental shift from social reciprocity to instrumental command—it requires a distinct pedagogical role. We propose a strategic division of labor based on Goffman’s dramaturgical theory [12]. AI should be legitimized as the “Cognitive Backstage”—a private, zero-friction rehearsal space for drafting, rapid iteration, and error-correction where social lubricants are unnecessary and efficiency is paramount.

However, this positioning elevates the importance of the human classroom as the “Cognitive Frontstage.” Rather than mourning the loss of politeness in AI interactions, educators should intentionally design the classroom to cultivate the high-friction collaborative skills that AI cannot simulate. If AI handles the low-friction acquisition of information, the classroom must become the primary site for negotiation, persuasion, and the management of high-stakes interpersonal feedback. Assessments should require students to move their AI-generated outputs from the “Backstage” to the “Frontstage,” forcing them to defend their ideas using social protocols of evidence and debate. The goal is to ensure that “Social Bypassing” remains a tool for efficiency, while social resilience remains the core currency of the human learning environment.

### Pedagogical and practical implications

The observed shift from social negotiation to “social bypassing” necessitates a pedagogical paradigm shift in how educators integrate GenAI into curricula. The implications of this study extend to three primary domains: instructional design, affective regulation, and the cultivation of GenAI literacy.

First, regarding instructional design, educators must transition from evaluating purely informational outputs to assessing the socio-cognitive processes of learning. Since AI effectively absorbs the low-friction “Cognitive Backstage” tasks (e.g., rapid error correction and syntax generation), classroom activities must be deliberately redesigned to amplify the “Cognitive Frontstage.” This implies deploying flipped-classroom models where students utilize AI for preparatory troubleshooting, but classroom time is strictly reserved for high-friction collaborative activities—such as peer code-reviews, Socratic questioning, and defending methodological choices—which AI cannot simulate.

Second, recognizing the “affective neutrality” and polarizing behavioral shifts (the “Director” vs. the “Confessor” stances) is critical for emotional regulation in learning environments. While interacting with AI minimizes immediate evaluation anxiety, it deprives learners of the emotional labor required to build academic resilience. Educators should be aware that excessive reliance on frictionless AI environments may leave vulnerable students ill-equipped to handle the constructive criticism inherent in human peer networks. Therefore, pedagogical interventions should explicitly bridge this emotional labor gap, fostering communities of practice where “failing publicly” is normalized rather than penalized.

Finally, our findings enrich the evolving concept of “GenAI literacy” [35]. Beyond technical prompt engineering, true GenAI literacy must encompass socio-emotional boundary management. Learners must be explicitly taught to recognize when to leverage AI for rapid product generation and when it is developmentally crucial to engage in the vulnerable, high-friction process of human inquiry. Institutional guidelines should not merely police academic integrity but must guide students in navigating the epistemic and social trade-offs of algorithm-mediated learning.

### Limitations

First, the comparison contrasts two distinct interaction modalities with differing governance structures. The high density of defensive linguistic markers in Stack Exchange is partially attributable to platform-specific enforcement (e.g., moderation rules), which acts as a confounding variable to pure social anxiety. Second, the dataset is cross-sectional and text-based; it does not track individual learning outcomes or cognitive retention over time. The inference regarding ‘cognitive bypassing’ is derived from behavioral traces rather than cognitive testing. Finally, a significant limitation lies in the potential demographic and expertise mismatch between the two corpora. Stack Exchange is a meritocratic community where hierarchies are explicit, and the “Petitioner” stance may be reinforced by the technical novice-to-expert dynamic. In contrast, the LMSYS dataset likely contains a broader spectrum of users, including experts using AI for efficient task offloading (e.g., generating boilerplate code). For such experts, the “Director” stance may reflect functional efficiency rather than a psychological response to reduced social friction. While our use of Propensity Score Matching and Topic Alignment partially mitigates this by comparing queries of similar complexity, the universality of the “Petitioner-Director” shift should be interpreted with caution across different expertise levels. Furthermore, it is important to distinguish between communicative choice and cognitive capacity. The observed shift toward “Product Generation” in AI interactions may reflect a rational preference for efficiency rather than an inability to engage in “Process Inquiry.” Because our study is cross-sectional and relies on behavioral traces (text) rather than pre- and post-testing of cognitive skills, we cannot conclude that users’ underlying abilities are being eroded. We present the potential for reduced practice as a theoretical risk rather than an empirically proven outcome.

## Conclusions

### Beyond preference: The structural transformation of inquiry

Ten years ago, the primary debate in educational psychology centered on whether students preferred the “warmth” of face-to-face interaction or the “convenience” of computer-mediated communication. Our investigation into large-scale conversational traces suggests that Generative AI has rendered this binary obsolete. The shift we observe is not merely a change in preference, but a fundamental restructuring of the interactional and epistemic economy. Our findings demonstrate a profound transformation that occurs when moving from a rule-bound, meritocratic public sphere to a private, frictionless command interface. In human communities, the necessity to pay a “politeness tax,” construct elaborate narratives of effort, and perform humility is a mandatory response to the combined pressures of social evaluation and platform governance. By removing these multi-layered constraints, Generative AI has created a frictionless “command economy” of information, where the learner transitions instantly from a petitioner seeking help to a director assigning tasks. This transition highlights how human sociality is contingent upon the very frictions—both psychological and institutional—that AI is designed to eliminate.

### The reality of “social bypassing”

Our findings fundamentally challenge the “Benign Disinhibition” hypothesis in educational contexts. The appeal of AI agents does not merely stem from their ability to foster psychological “safe vulnerability”—indeed, learners rarely admit ignorance to the machine. Rather, the appeal lies in Social Bypassing. AI is seductive because it grants epistemic privilege: the power to acquire knowledge without the cognitive costs of impression management and the bureaucratic overhead of complying with human community norms. The shift to AI represents a transition from a highly regulated, institutionally governed social space to a purely functional, frictionless interface. It allows learners to be “wrong” without ever having to acknowledge their error to a judging peer. While this efficiency is attractive, it represents a form of socially detached efficiency rather than genuine psychological safety.

### Preserving the “high-friction” classroom

As we integrate AI into education, we face the risk of conflating “efficiency” with “learning.” The “Director” stance, while effective for rapid information retrieval, highlights a potential risk regarding the long-term development of soft skills required for collaborative inquiry. Our findings suggest that habituation to a compliant, judgment-free environment could theoretically reduce the opportunities to practice the resilience needed for real-world negotiation. Future longitudinal research is required to determine whether this behavioral shift leads to a measurable decline in cognitive and social competencies.

Therefore, the future of educational design relies on a clear demarcation of spaces. We must legitimize AI as the “Cognitive Backstage”—a private space for drafting and error correction. However, we must simultaneously vigorously defend the human classroom as the “Cognitive Frontstage”—a space intentionally designed for high-friction debate, where students must defend their ideas using the very social protocols that AI allows them to bypass.

### Final thought

Ultimately, Generative AI is an unmatched engine for answering questions, but it is a poor substitute for the social process of questioning itself. The goal of education is not simply to reduce the anxiety of not knowing, but to teach students how to navigate that anxiety within a community of peers. As the landscape of Artificial Intelligence continues to expand across diverse specialized domains—from cybersecurity and phishing detection [55] to medical classification [56] and automated hardware systems [57]—the fundamental role of human-centered social inquiry becomes even more pronounced. We must ensure that in our pursuit of algorithmic convenience, we do not inadvertently reduce the opportunities for students to engage in the essential cognitive and social frictions that have traditionally structured the learning process.
